# Supplementary figures and images for: Endogenous tagging using split mNeonGreen in human iPSCs for live imaging studies
Source: eLife. 2024 Apr 23;12:RP92819. doi: 10.7554/eLife.92819 (PMC11037917; doi:10.7554/eLife.92819)

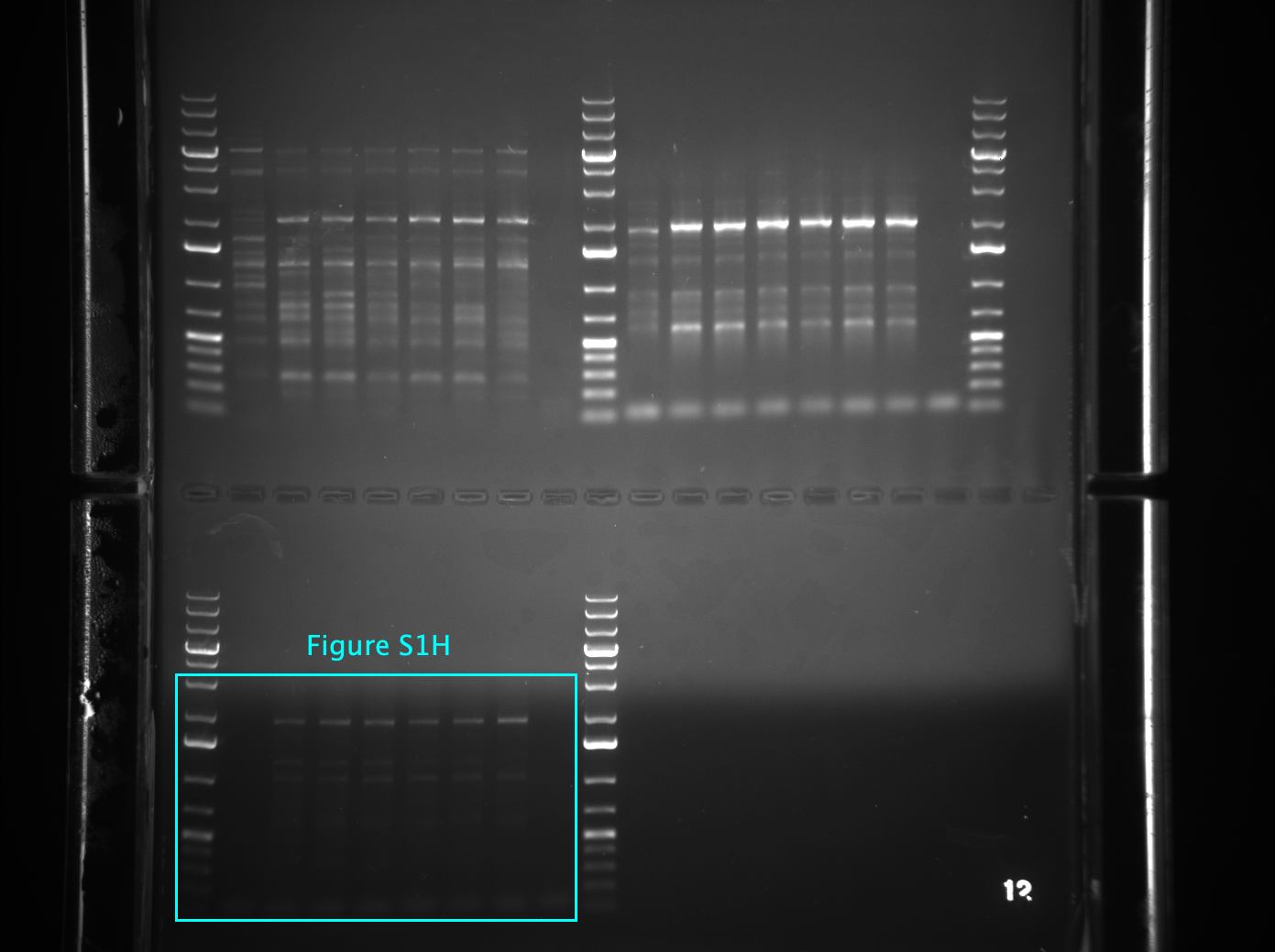

Supplement: Figure 1—figure supplement 1—source data 2. [file elife-92819-fig1-figsupp1-data2.zip › Figure 1-figure supplement 1-Source Data 2/Figure 1-figure supplement 1H-Annotated gel image.tif]

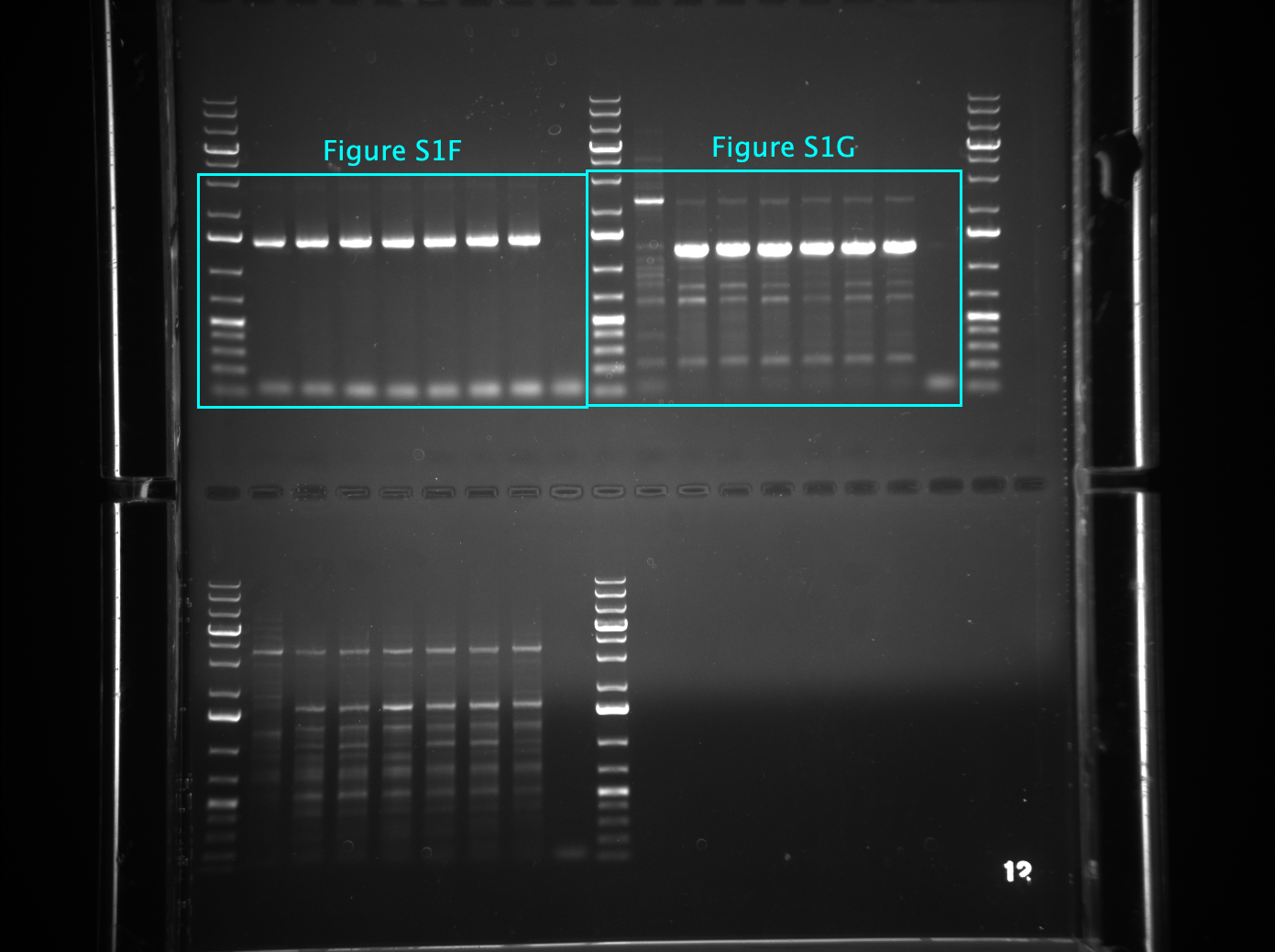

Supplement: Figure 1—figure supplement 1—source data 2. [file elife-92819-fig1-figsupp1-data2.zip › Figure 1-figure supplement 1-Source Data 2/Figure 1-figure supplement 1F and G-Annotated gel image.tif]

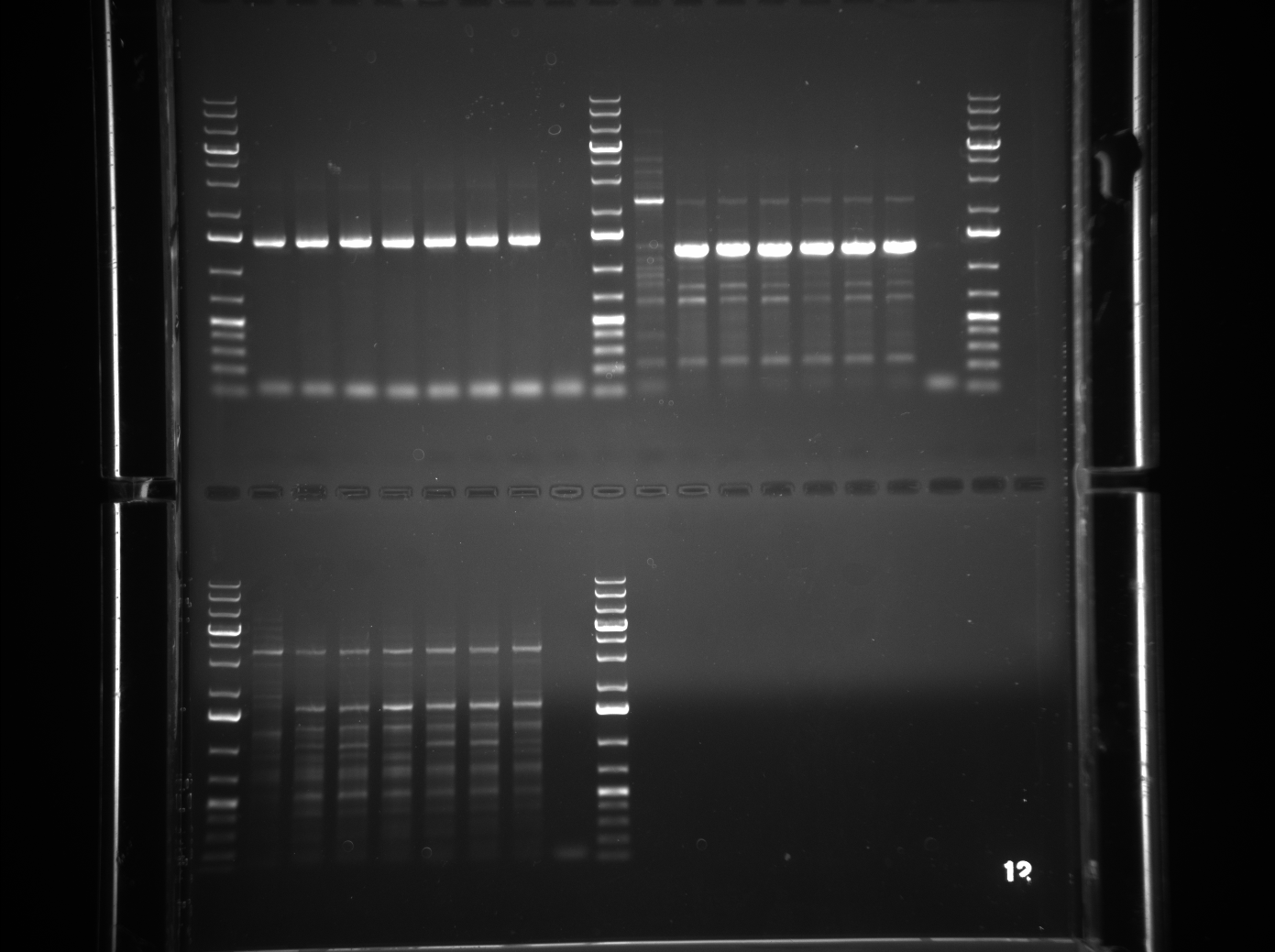

Supplement: Figure 1—figure supplement 1—source data 2. [file elife-92819-fig1-figsupp1-data2.zip › Figure 1-figure supplement 1-Source Data 2/Figure 1-figure supplement 1F and G-Raw gel image.tif]

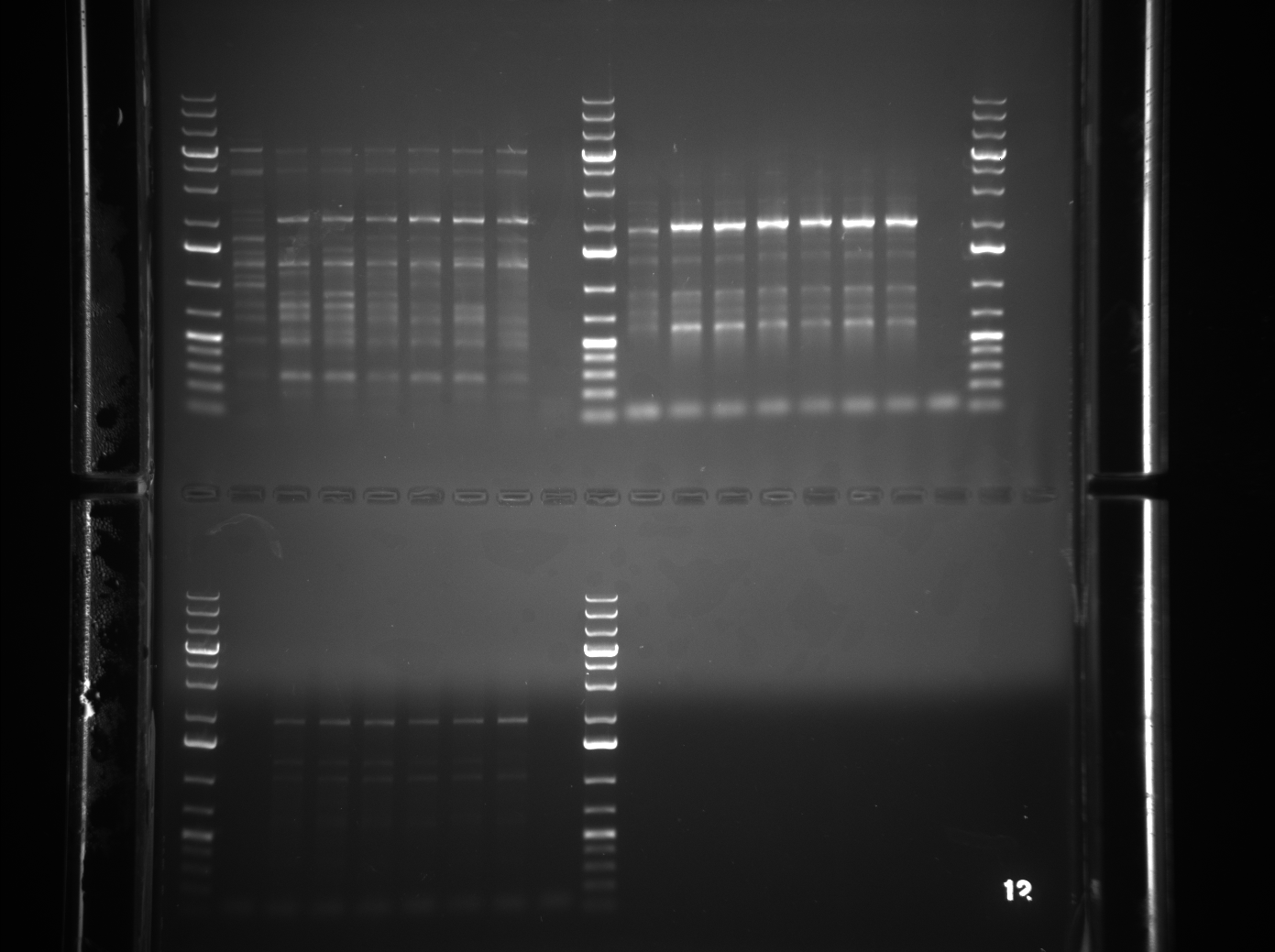

Supplement: Figure 1—figure supplement 1—source data 2. [file elife-92819-fig1-figsupp1-data2.zip › Figure 1-figure supplement 1-Source Data 2/Figure 1-figure supplement 1H-Raw gel image.tif]

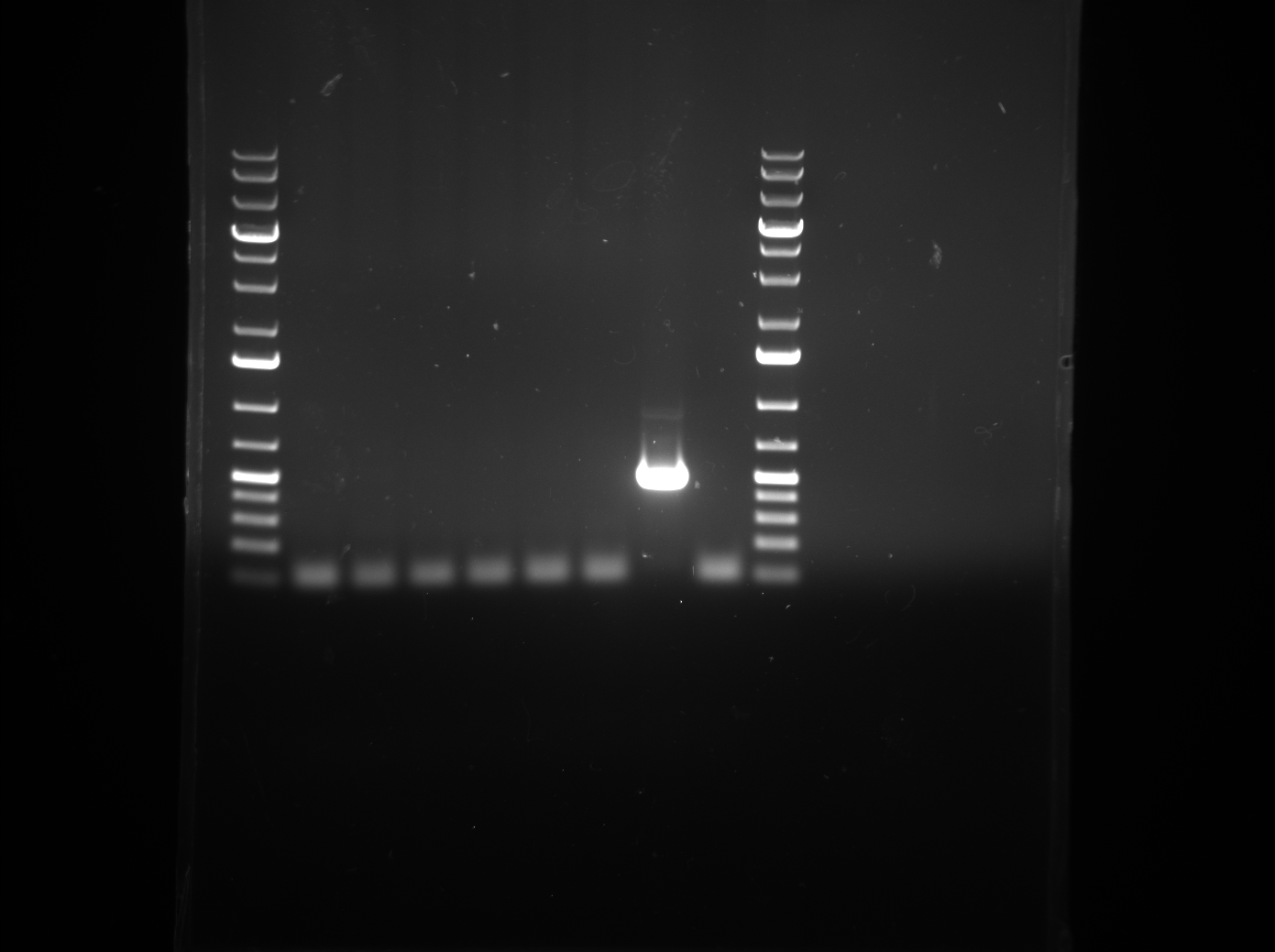

Supplement: Figure 1—figure supplement 2—source data 2. [file elife-92819-fig1-figsupp2-data2.zip › Figure 1-figure supplement 2-Source Data 2/Figure 1-figure supplement 2O-Raw gel image.tif]

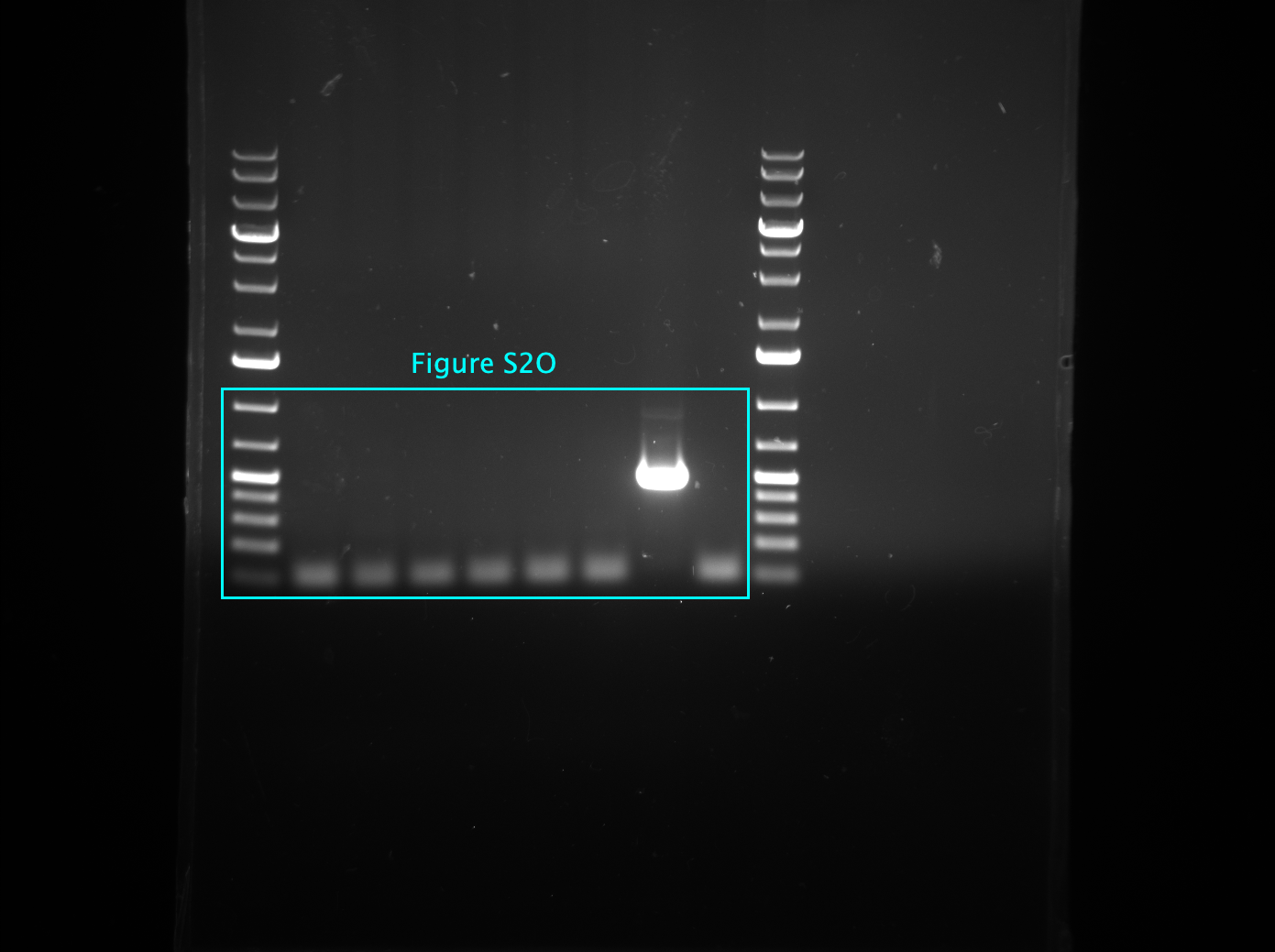

Supplement: Figure 1—figure supplement 2—source data 2. [file elife-92819-fig1-figsupp2-data2.zip › Figure 1-figure supplement 2-Source Data 2/Figure 1-figure supplement 2O-Annotated gel image.tif]

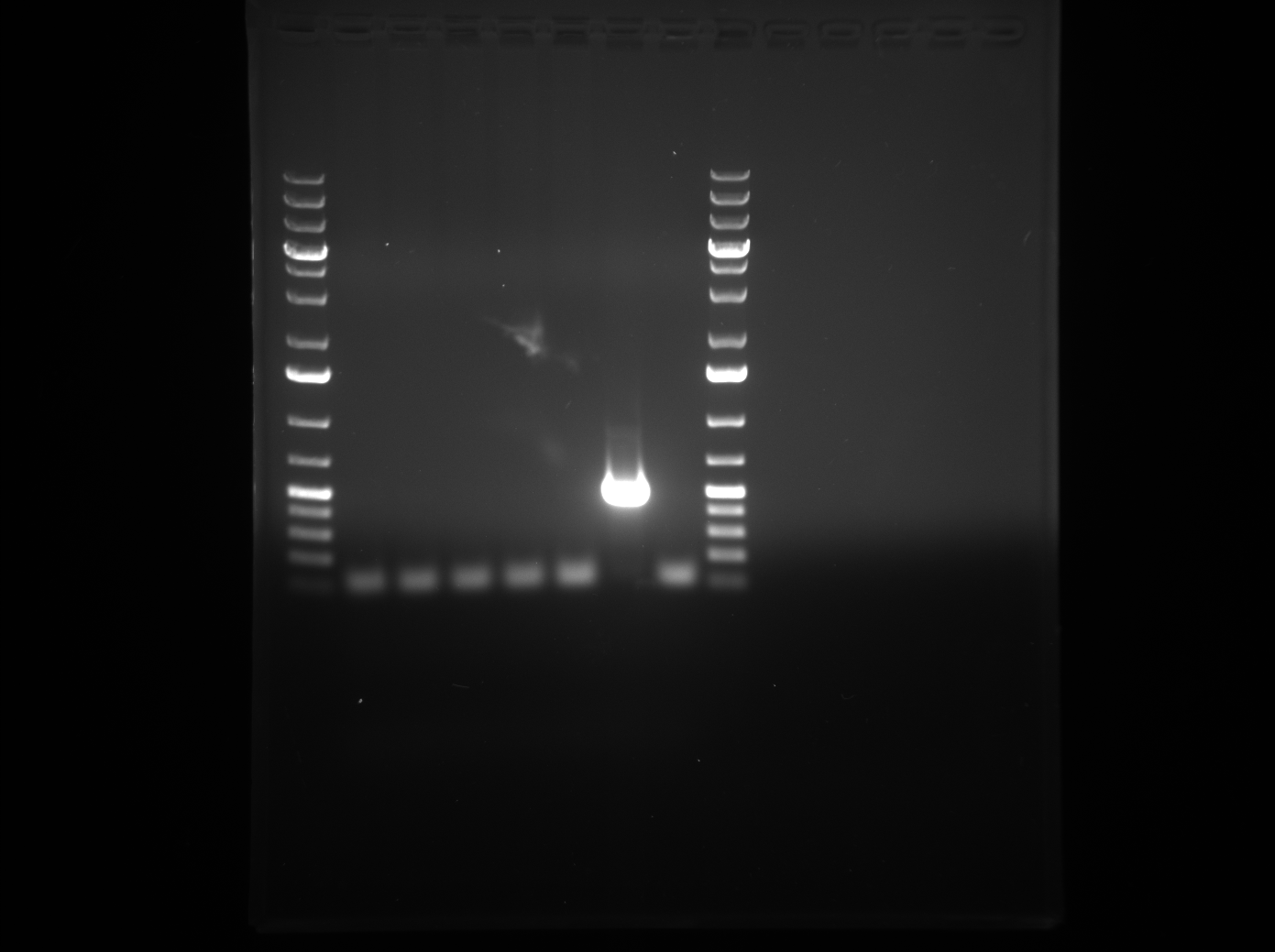

Supplement: Figure 3—figure supplement 1—source data 2. [file elife-92819-fig3-figsupp1-data2.zip › Figure 3-figure supplement1-Source Data 2/Figure 3-figure supplement 1J-Raw image of the first gel.tif]

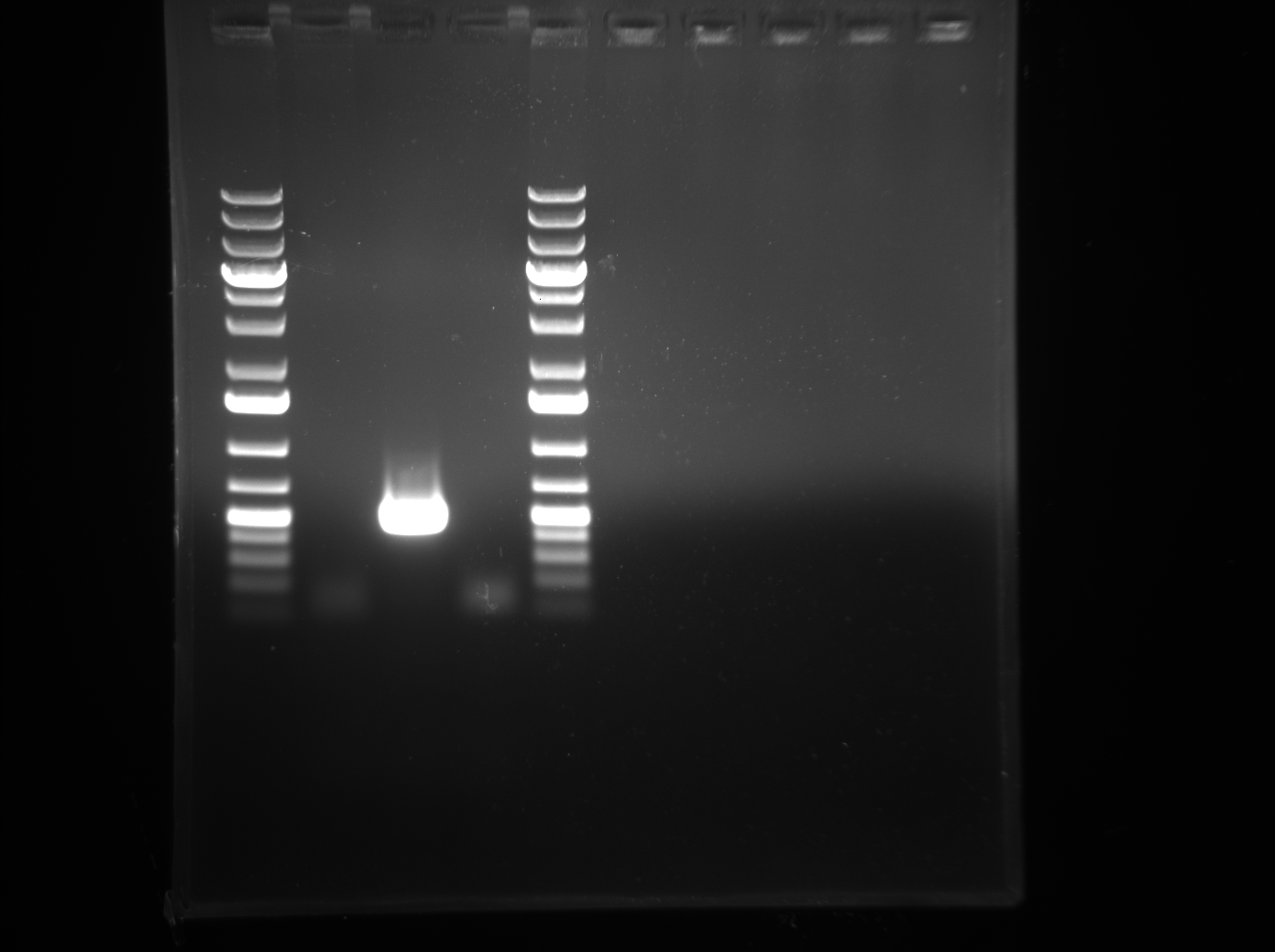

Supplement: Figure 3—figure supplement 1—source data 2. [file elife-92819-fig3-figsupp1-data2.zip › Figure 3-figure supplement1-Source Data 2/Figure 3-figure supplement 1J-Raw image of the second gel.tif]

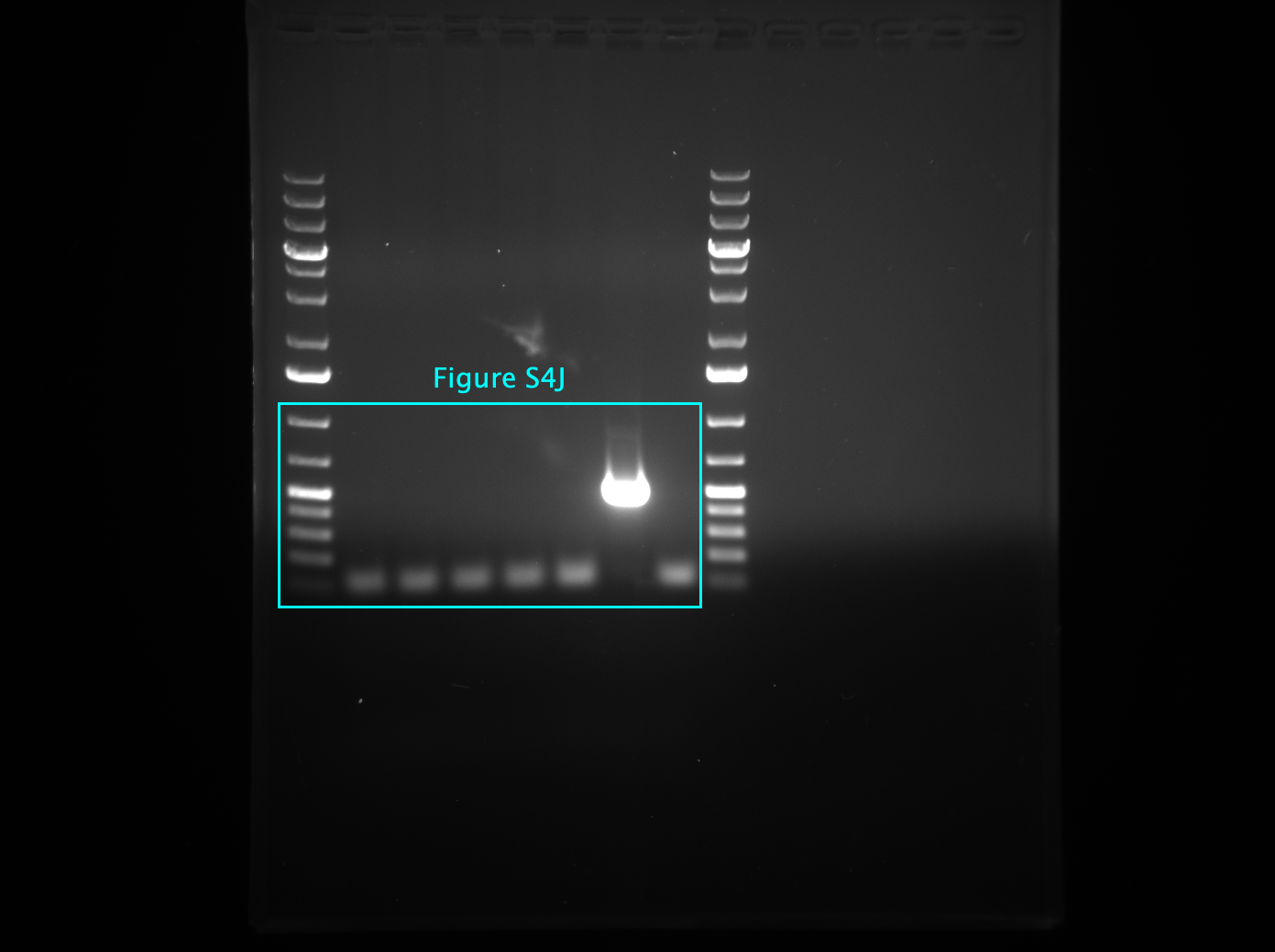

Supplement: Figure 3—figure supplement 1—source data 2. [file elife-92819-fig3-figsupp1-data2.zip › Figure 3-figure supplement1-Source Data 2/Figure 3-figure supplement 1J-Annotated image of the first gel.tif]

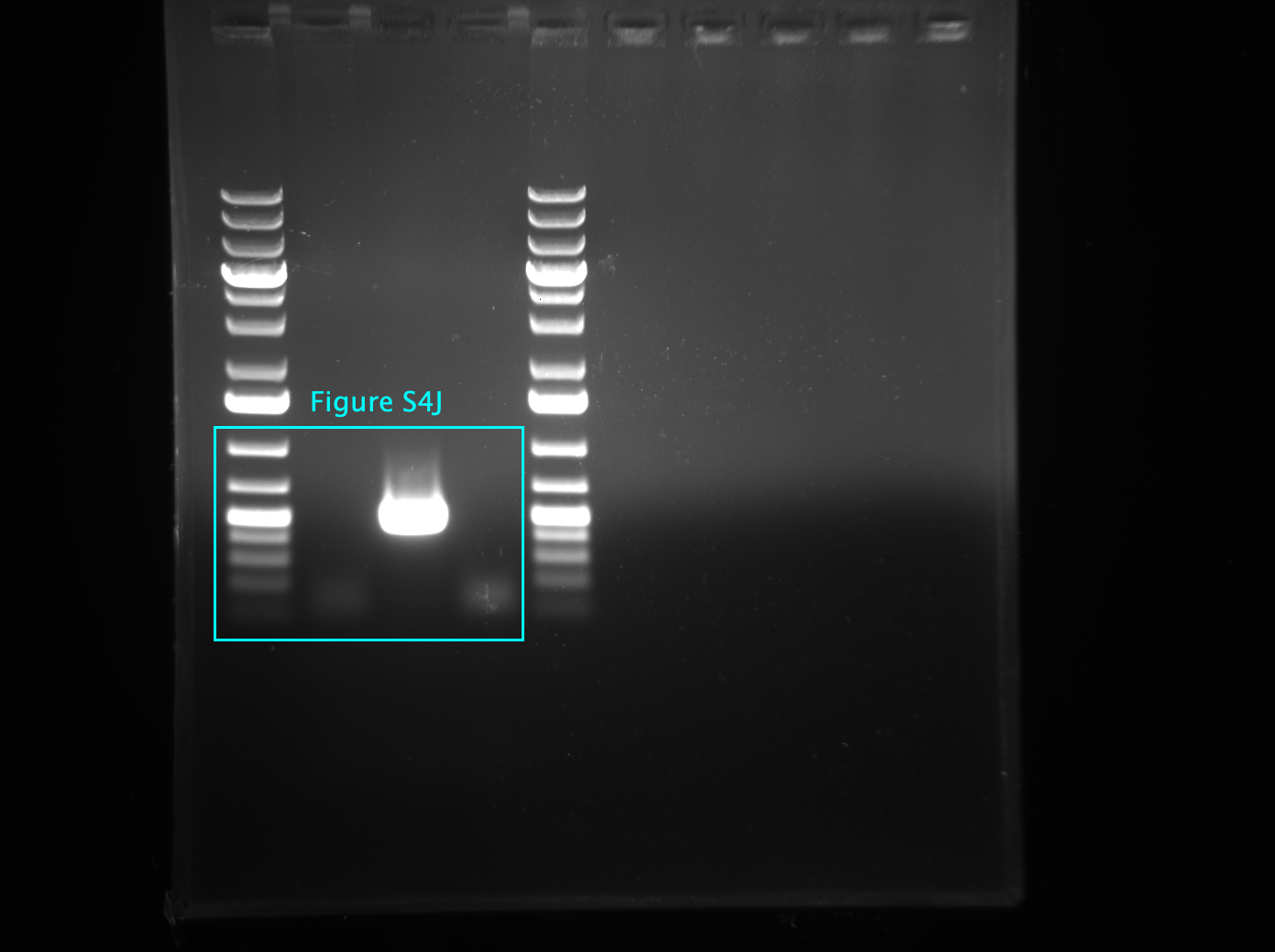

Supplement: Figure 3—figure supplement 1—source data 2. [file elife-92819-fig3-figsupp1-data2.zip › Figure 3-figure supplement1-Source Data 2/Figure 3-figure supplement 1J-Annotated image of the second gel.tif]
